# Transcriptome Analysis of *Hamelia patens* (Rubiaceae) Anthers Reveals Candidate Genes for Tapetum and Pollen Wall Development

**DOI:** 10.3389/fpls.2016.01991

**Published:** 2017-01-06

**Authors:** Lin Yue, David Twell, Yanfeng Kuang, Jingping Liao, Xianqiang Zhou

**Affiliations:** ^1^Key Laboratory of Plant Resources Conservation and Sustainable Utilization, South China Botanical Garden, Chinese Academy of SciencesGuangzhou, China; ^2^College of Life Sciences, University of Chinese Academy of SciencesBeijing, China; ^3^Department of Genetics, University of LeicesterLeicester, UK; ^4^Beijing Genomic InstituteShenzhen, China

**Keywords:** transcriptome, Rubiaceae, *Hamelia patens*, anther, tapetum, pollen wall

## Abstract

Studies of the anther transcriptome on non-model plants without a known genome are surprisingly scarce. RNA-Seq and digital gene expression (DGE) profiling provides a comprehensive approach to identify candidate genes contributing to developmental processes in non-model species. Here we built a transcriptome library of developing anthers of *Hamelia patens* and analyzed DGE profiles from each stage to identify genes that regulate tapetum and pollen development. In total 7,720 putative differentially expressed genes across four anther stages were identified. The number of putative stage-specific genes was: 776 at microspore mother cell stage, 807 at tetrad stage, 322 at uninucleate microspore stage, and the highest number (1,864) at bicellular pollen stage. GO enrichment analysis revealed 243 differentially expressed and 108 stage-specific genes that are potentially related to tapetum development, sporopollenin synthesis, and pollen wall. The number of expressed genes, their function and expression profiles were all significantly correlated with anther developmental processes. Overall comparisons of anther and pollen transcriptomes with those of rice and Arabidopsis together with the expression profiles of homologs of known anther-expressed genes, revealed conserved patterns and also divergence. The divergence may reflect taxon-specific differences in gene expression, the use RNA-seq as a more sensitive methodology, variation in tissue composition and sampling strategies. Given the lack of genomic sequence, this study succeeded in assigning putative identity to a significant proportion of anther-expressed genes and genes relevant to tapetum and pollen development in *H. patens.* The anther transcriptome revealed a molecular distinction between developmental stages, serving as a resource to unravel the functions of genes involved in anther development in *H. patens* and informing the analysis of other members of the Rubiaceae.

## Introduction

Pollen grains are the microgametophytes of seed plants that produce the male gametes required for sexual reproduction (Borg and Twell, 2011). Although the overall structure of pollen is conserved in angiosperms, pollen shows considerable variation in size, shape, and surface characteristics (Heslop-Harrison, 1968; Blackmore et al., 2007). Recently, the diversity and evolution of palynological characters were documented across the angiosperms based on the most comprehensive classification APG III (2009). Many characters, such as exine structure and aperture features, have indicated key evolutionary transitions in pollen morphology and have proven to be informative at different taxonomic levels (Lu et al., 2015; Wortley et al., 2015). However, little is known about the genetic basis underlying pollen evolution. Comparative analysis has indicated significant conservation in anther gene expression patterns between *Arabidopsis* and maize (Ma et al., 2008). Further comparative analysis of anther and pollen transcriptome profiles for diverse angiosperms would complement understanding the conservation of the molecular mechanisms underlying pollen development (Wilson and Zhang, 2009; Rutley and Twell, 2015).

Pollen development involves the coordination of cellular activities and the underlying gene expression in sporophytic and gametophytic cells of the anther (Ariizumi and Toriyama, 2011). Anthers are composed of developing gametophytes and a number of surrounding cell layers: the innermost tapetum, the ML, the endothecium, and the outer epidermis. Initially, archesporial cells form in the L2 layer of the anther primordium and divide periclinally to form outer primary parietal cells (PPCs) and inner primary sporogenous cells. The primary sporogenous cells undergo divisions to form MMCs, whereas the PPCs undergo a series of divisions to form the cell layers of the anther wall (Scott et al., 2004). The MMCs undergo meiosis producing tetrads of haploid microspores at tetrad stage (TET). The tetrads are usually separated as free microspores after the callosic walls are dissolved by callase at the UNM stage. The free microspores divide asymmetrically to segregate the male germline and develop further into PG before release at BCP stage or TCP stage (McCormick, 1993; Twell, 2011). Pollen development relies on the complex interactions between reproductive and non-reproductive tissues of the anther, especially the tapetum, which provides enzymes for the dissolution of tetrads, nutrients to the developing microspores and materials deposited onto the pollen exine (Goldberg et al., 1993; Tsuchiya et al., 1994; Li et al., 2006).

A significant number of key genes required for anther and pollen development have been identified by forward genetic screens in model and crop plants. These genes, which include a significant number of transcriptional regulators, control formation, and degeneration of the tapetum, microspore release from the tetrad and formation of the complex pollen wall (Boavida et al., 2005; Wilson and Zhang, 2009; Shi et al., 2015). Key genes involved in tapetum formation and differentiation include, *EXCESS MICROSPOROCYTES1/EXTRA SPOROGENOUS CELLS (EMS1/EXS)* (Zhao et al., 2002) and *TAPETUM DETERMINANT 1(TPD1)* (Yang et al., 2003). Genes involved in the programmed cell death (PCD) of tapetum cells include *TAZ1* (Kapoor et al., 2002), *TAPETUM DEGENERATION RETARDATION (TDR)* (Li et al., 2006), *PERSISTENT TAPETAL CELL 1 (PTC1), API5* (Li et al., 2011), *Osc6* (Zhang et al., 2010), and *CEP1* (Zhang D.D. et al., 2014). The absence of these genes results in abnormal tapetal PCD and sterile pollen.

Transcriptional regulation is a major mechanism controlling anther development in *Arabidopsis*. The bHLH transcription factor (TF) *DYSFUNCTIONAL TAPETUM1 (DYT1)* plays a critical role in regulating tapetum function and pollen development (Zhang et al., 2006; Feng et al., 2012; Zhu et al., 2015). DYT1 regulates the expression of the *ABORTED MICROSPORES (AMS)* (Sorensen et al., 2003; Xu et al., 2014), *MALE STERILITY1 (MS1)* (Vizcay-Barrena and Wilson, 2006), and other tapetum preferential genes, primarily via *DEFECTIVE IN TAPETAL DEVELOPMENT AND FUNCTION 1* (*TDF1*) (Gu et al., 2014). Important regulators of tetrad callose degradation include the *Arabidopsis* TF gene *AtMYB103* (Higginson et al., 2003; Zhang et al., 2007), and the rice β-1,3-glucanase encoded by *Osg1* (Wan et al., 2011). Key genes required for pollen exine development have also been characterized, including *MS2* (Aarts et al., 1997), *ACOS5* (Souza et al., 2009), *CYP703A2* (Morant et al., 2007), *CYP703A3* (Aya et al., 2009), and *EFD* (Hu et al., 2014). Moreover, recent co-expression analysis revealed that AMS acts as a master regulator coordinating pollen wall development and sporopollenin biosynthesis in *Arabidopsis* (Xu et al., 2014). Thus, current understanding of anther regulatory genes is overwhelmingly derived from studies of model plants and crops and little information about gene activities in developing anthers is known from non-model species.

Transcriptome analyses have significantly enriched knowledge of the repertoire of genes expressed during pollen development. Genome-scale analyses have been described for developing anthers and pollen, germinating pollen and pollen tubes, and mature pollen transcriptome data for at least 10 different species (Huang et al., 2011; Rutley and Twell, 2015). Transcriptome profiling at different developmental stages have been reported for *Arabidopsis* (Honys and Twell, 2004), rice (Wei et al., 2010), and tobacco (Bokvaj et al., 2015). Transcriptome studies of developing *Arabidopsis* anthers and pollen have revealed genes that function as early as meiosis, in the tapetum and in exine formation (Dukowic-Schulze and Chen, 2014). Recently, genome-wide co-expression analysis revealed 98 candidate genes closely associated with pollen wall development (Xu et al., 2014). Moreover, comparative studies in rice involving microarray studies of developing anthers have also revealed complex transcriptomes (Deveshwar et al., 2011).

RNA-Seq based methods have led to a dramatic acceleration in gene discovery (Barrero et al., 2011; Garg et al., 2011; Shi et al., 2011), which has rapidly broadened understanding of the complexity of gene networks and their regulation (Wang et al., 2010; Hua et al., 2011). Moreover, there are limitations of microarray technology such as lack of quantitative detection of transcripts with different probe sets, low abundance transcripts are difficult to detect, and a reference genome and/or expressed sequence tags (ESTs) are required (Huang et al., 2011; Zhao et al., 2014). Studies of anther transcriptomes based on RNA-seq are limited to those in a few crops and model plants, however, expression profiling of developing anthers by Next Generation Sequencing (NGS) from species whose genomes have not been sequenced is anticipated (Wang et al., 2009; Huang et al., 2011).

Rubiaceae, commonly known as the coffee family, is the fourth largest in the angiosperms and has important economic utilities; in addition to the valuable beverage crop coffee, as a source of quinine, dyes, and ornamentals. It is a eurypalynous family with great diversity in pollen size and shape, aperture type, number and position, pollen wall stratification, and sexine ornamentation (Dessein et al., 2005). Some pollen features have significant value in stimulating further palynological investigations, for example, the development of Ubisch bodies provides a potential model for studying sporopollenin deposition (Dessein et al., 2005). However, there is limited information about the gene expression patterns underlying anther and/or pollen development in Rubiaceae. Current molecular data from model species and a few crop plants are insufficient to resolve the mechanisms that regulate the development, function, and evolution of angiosperm pollen. More attention to such a palynologically well-documented family would help clarify the molecular processes underlying anther/pollen development in angiosperms and the associated evo-devo mechanisms.

In this paper, *Hamelia patens* Jacq. was chosen for study as a member of the coffee family. *H. patens* originates in tropical America and is a popular ornamental plant in south and southwest China. The ease of cultivation and long blooming period from May to October enables facile collection of floral material. Further, this species produces triaperturate PG, representing the plesiomorphic condition in the Rubiaceae family. These advantages make *H. patens* a potentially valuable system for developmental and molecular research in Rubiaceae.

Transcriptome profiles of *Hamelia patens* developing anthers from MMC to BCP grain stages were investigated using NGS and NGS-based digital gene expression (DGE) tag profiling. The aims of the present study were, (1) to provide molecular insight into anther and pollen development of this phylogenetically and palynologically important family, and (2), to reveal novel candidate genes involved in tapetum and pollen development, to shed light on the evo-devo mechanisms of angiosperm pollen development.

## Materials and Methods

### Sample Preparation for Anther Development

Flowers of *Hamelia patens* Jacq. at various developmental stages were harvested from plants growing in South China Botanical Garden (SCBG), Guangdong Province, China. Each flower contains five anthers. The length of flowers and the anthers was measured using a stereo microscope. The classification of flowers and anthers for categorization of anther developmental stages is summarized in **Table 1**.

**Table 1:** Classification of flowers and anthers for categorization of anther developmental stages.

Pollen developmental stage	Flower length (cm)	Anther length (cm)
Microspore mother cell stage	0.30–0.80	0.20–0.45
Tetrad stage	0.90–1.00	0.50–0.60
Uninucleate microspore stage	1.10–1.60	0.60–0.85
Bicellular pollen stage	1.80–2.70	0.91–1.01

The anthers were fixed in 2.5% glutaraldehyde in 0.1 mol/L phosphate buffer, pH 7.2, placed under a vacuum for 2 h, then stored at 4°C for several days. After removal from storage, the anthers were rinsed in 0.1 mol/L phosphate buffer for 2 h, and post-fixed in 1% osmium tetroxide overnight. Following post-fixation, anthers were washed in phosphate buffer, dehydrated in an acetone series, embedded in PON-812 resin and cured at 70°C. Semi-thin sections (1–2 μm) were cut with glass knives on a LKB-11800 microtome, stained with 0.1% Toluidine Blue, and observed and photographed with a Leica – DM5500B light microscope (LeiCa, Germany). Ultrathin sections (80 nm) were cut using a Leica-Ultracut S ultramicrotome with a diamond knife, and stained with uranyl acetate and lead citrate. Transmission electron micrographs were taken with a JEOL JEM-1010 (JEOL, Japan) transmission electron microscope at 100 KV.

For SEM, the PG were mounted on copper stubs with a strip of double-sided conductive tape and air-dried. The sample was then coated with gold in a JEOL JFC-1600 sputter coater. Observations and digital images were collected with a JEOL JSM-6360LV SEM. Measurements of the polar axis (P) and equatorial diameter (E) were made on digital SEM images with JEOL’s Smile View software.

### Sample Preparation for RNA Extraction

Transcriptome profiling was carried out on developing anthers at four landmark stages: MMC stage, tetrad (TET) stage, UNM stage, and BCP stage. The four stages were defined on the basis of cytological observations with LM and TEM as described above and confirmed using DAPI staining.

Fresh flowers were collected from three well-managed populations of *H. patens* in the SCBG. The anthers were picked out under a dissecting microscope, immediately frozen in liquid nitrogen and stored at -80°C until RNA extraction. They were classified into four separate groups according to length and each group was pooled by mixing equal quantities of anthers from the three populations.

Total RNA was extracted using pBIOZOL method. RNA quality was characterized by using a NanoDrop ND1000 spectrophotometer (NanoDrop, USA), and by determination of the RIN (RNA Integrity Number) value (≥7.3) using an Agilent 2100 Bioanalyzer.

### Library Preparation and Sequencing

The cDNA libraries were prepared following manufacturer’s instructions (Illumina, USA). Five cDNA libraries (MMC, TET, UNM, BCP, and MSA) were established. MSA was a mixed stage anther sample containing equal amounts of anthers from each of the four stages (MMC, TET, UNM, and BCP). mRNA was enriched using oligo (dT) magnetic beads from 5 μg total RNA (total RNA amount of MSA was 20 μg). To avoid priming bias during cDNA synthesis, isolated mRNAs were first fragmented into short pieces (about 200 bp) using RNA Fragmentation Reagents (Ambion, USA). The cleaved mRNA fragments were converted to double-stranded cDNA using random hexamer primers (Illumina) with the SuperScript Double-Stranded cDNA Synthesis kit (Invitrogen, USA). The double-stranded cDNA was purified using a QiaQuick PCR Purification kit (Qiagen, USA) and was processed by end-repair using End-Repair Mix Reaction System (Beijing Genomic Institute, China) and the addition of a single adenine. The repaired cDNA fragments were then ligated with sequencing adapters. To select a size range of templates for downstream enrichment, the products of the ligation reaction were purified on a 2% TAE-agarose gel. Then the purified cDNA was enriched by PCR amplification using Primer PE 1.0 and PE 2.0 (Illumina) complementary to the ends of the adapters with Phusion DNA Polymerase. The library products were then sequenced using Illumina HiSeq^®^ 2000 at the Beijing Genomic Institute, China.

### Data processing and *De novo* Assembly

The original data produced by the sequencer were defined as raw reads. Base calling accuracy was measured by the Phred quality score (Q score), which is the most common metric used to assess the accuracy of a sequencing platform. Q20 stands for 99% accuracy representing probability of incorrect base call being 1 in 100, while Q30 stands for 99.9% accuracy representing probability of incorrect base call being 1 in 1000 (Illumina technique notes: sequencing). Filtering of raw reads was carried out to obtain clean reads by remove those, (a) containing adaptors, (b) containing more than 5% unknown nucleotides, and (c) showing more than 10% of bases with a Phred scaled probability (Q) less than 20.

Clean reads were assembled into unigenes using the short reads assembly program Trinity, release-20130225^1^. The resulting unigenes were divided into two major classes. One class containing unigenes that had a similarity of more than 70%, and a singletons class (Iseli et al., 1999; Grabherr et al., 2011).

### Functional Annotation and Analysis

To assign putative gene function, unigene sequences were firstly aligned by BLASTx (*E*-value < 1e-5) to protein databases (NR, SwissProt, KEGG, COG). Then the unigenes were searched against nucleotide database NT (*E*-value < 1e-5) using BLASTx to retrieve proteins with the highest sequence similarity with the given unigenes and their protein functional annotations. Functional categories of the predicted genes were obtained by applying gene ontology (GO) terms to the NR database annotation using the Blast2GO program. GO functional classification for all unigenes and the distribution of gene functions were analyzed using WEGO software (Conesa et al., 2005; Ye et al., 2006). To identify possible functions, the annotated unigene sequences were searched in the COG database where orthologous gene products are classified (Kanehisa et al., 2008). To identify active pathways in *H. patens* anthers, annotated sequences were mapped to reference pathways in the KEGG database (Release 63.0). COG and KEGG pathway annotations were performed using BLASTall software.

### Comparison with the Coffee Genome

Since *Coffea canephora* is the only species in the Rubiaceae with a sequenced genome, comparison analysis was conducted between the unigenes of *H. patens* and the *C. canephora* CDSs using BLASTn. Sequence similarity was estimated by the coverage of the matched length, by calculating the value of matched sequence length versus unigene length and CDS length, respectively. The coffee genome was downloaded from the following website^2^. Moreover, the expression level and the GO annotations were examined on a set of genes with relatively high sequence similarity to their orthologs in coffee.

### Digital Gene Expression Tag Profiling Analysis

In order to predict putative genes correlating with tapetum and pollen development, four separate DGE libraries (MMC, TET, UNM, and BCP) were established as mentioned above and analyzed. Procedures of library construction and sequencing were the same with the MSA library. All clean reads from the four libraries were mapped onto the transcriptome library (MSA) to calculate the expression level for each gene and the gene coverage using SOAP (version 2.21). If there was more than one transcript for a gene, the longest one was used to calculate its expression level and coverage. The gene expression level was calculated using RPKM value. The gene coverage was determined as the ratio of the base number in a gene covered by unique mapping reads to the total bases number of the gene. RPKM value ≥ 10 was used as a criterion to define high-level expressed genes. RPKM value of 10 is the average of the median for every gene plus 5. Highly expressed genes were categorized with a Venn diagram.

Digital Gene Expression was used to compare the differences in gene expression (Audic and Claverie, 1997). The threshold FDR < 0.001 and the absolute value of the log_2_Ratio ≥ 1 were used to determine the potential difference in gene expression. Genes enriched at a specific stage were identified by comparing gene expression between two adjacent stages. Genes that were twofold up-regulated and expressed at high-level (RPKM ≥ 10) at the same stage were identified. Differentially expressed genes (DEGs) were twofold up-regulated at only one stage or simultaneously at two stages or three stages, which were identified by pairwise comparison of the four stages of anther development. Among the twofold up-regulated genes at only one stage, those with high expression (RPKM value ≥ 10) at the same stage and meanwhile with low expression (RPKM value ≤ 10) at the other three stages were defined as specifically expressed genes (SEGs). Then we carried out GO functional enrichment and KEGG pathway enrichment analysis for these SEGs. Cluster analysis of gene expression patterns was performed with Cluster (De Hoon et al., 2004) and Java Treeview (Saldanha, 2004) softwares. With GO enrichment analysis, first all DEGs were mapped to GO terms in the database^3^, then the gene numbers for every term was calculated, hypergeometric test was used to find significantly enriched GO terms in DEGs. The calculated *p*-value went through Bonferroni Correction, taking corrected *p*-value ≤ 0.05 as a threshold.

With Nr annotation, Blast2GO program was used to get GO annotation of DEGs, and then WEGO software (Ye et al., 2006) was used to do GO functional classification for DEGs. KEGG pathway enrichment analysis was used to identify significantly enriched metabolic pathways or signal transduction pathways in DEGs (Kanehisa et al., 2008).

### Real-Time Quantitative PCR (q-PCR)

qRT-PCR was performed on independently collected samples to verify the potentially differentially expressed genes (DEGs) identified by RNA-seq. Total RNA was prepared from *H. patens* anthers at the four developmental stages as the same for DGE analysis. First-strand cDNA was synthesized using a FastQuant RT Kit (TIANGEN, China) according to the manufacturer’s protocol. Real-time PCR primer was designed on Web Primer: DNA and Purpose Entry website^4^. Primers used in the experiment are listed in **Supplementary Table S1**. All reactions were performed using GoTaq qPCR Master Mix (Promega, USA). Reactions were carried out in a total volume of 10 μL reaction mixture containing 5.0 μL of GoTaq qPCR Master Mix (Promega), 0.2 μL (10 μmol/L) of each primer, 200 ng of template cDNA. The real-time RT-PCR amplification was performed with LightCycler 480 || Real-Time PCR System using two-step cycling conditions of 95°C for 10 min, followed by 40 cycles of 95°C for 15 s and 60°C for 60 s. Dissociation stage condition was set at 95°C for 15 s, 60°C for 15 s, and 95°C for 15 s. The β-Actin primer was designed based on the homologous gene of *Rubia cordifolia* L. β-Actin was amplified from *H. patens* anthers at the same four stages and used as an internal control. The relative quantities of transcripts were calculated using the comparative Ct method and three biological replicates were performed.

## Results

### Ultrastructural Observations of Anther Wall and Pollen Development

**FIGURE 1:**
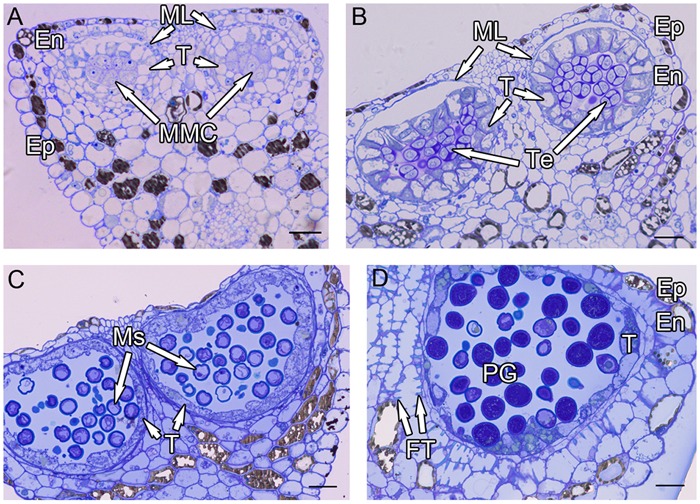
**Semi-thin sections of *Hamelia patens* anthers. (A)** Microspore mother cell stage, showing the cell layers of the anther wall and the microspore mother cells. **(B)** Tetrad stage, the tapetal cells become vacuolated, the ML flattened. **(C)** Uninucleate microspore stage, showing free microspores and cell layers of the anther wall. **(D)** Bicellular pollen stage, showing PG and FT in the endothecium. Scale bars in **(A–D)** represent 25 μm.

Anthers of *H. patens* contain four microsporangia. In cross sections, the MMCs are angular in shape and possess a large nucleus with a darkly staining Nu (**Figure 1A**). The anther wall at the MMC stage is differentiated into epidermis, endothecium, ML, and tapetum. The tapetum is uninucleate, one or two layered and the MMCs become oval prior to meiosis (**Figure 1A**).

**FIGURE 2:**
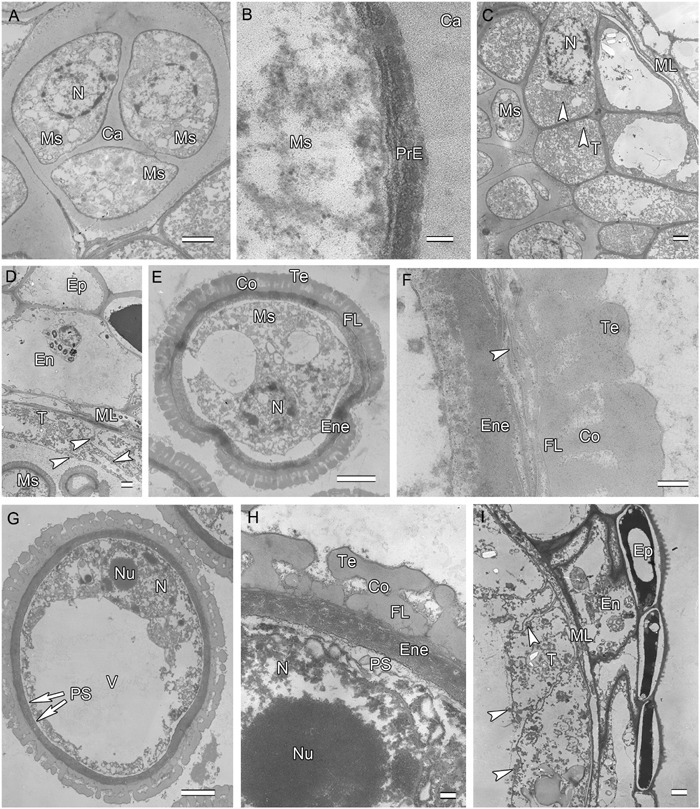
**Ultra-thin sections of *H. patens* anthers. (A)** Tetrad stage, the tetrads encased by a communal callose wall. **(B)** Tetrad stage, primexine matrix forms between the callose wall and the microspore. **(C)** Tetrad stage, showing fragmentation of nucleoli, small vesicles, and pro-Ubisch bodies (white arrowheads). **(D)** Uninucleate microspore stage, showing abundant Ubisch bodies produced in the tapetum (white arrowheads). **(E)** Showing a free microspore with differentiated wall. **(F)** Showing white-line-centered lamella (white arrowhead) between the FL and the endexine. **(G)** Vacuolated microspore stage, the nucleus is positioned against the cell wall. **(H)** Showing the PS formed beneath the exine. **(I)** Showing cell layers of the anther wall and Ubisch bodies in the tapetum (white arrowheads). Scale bars in **(A,C–E,G,I)** represent 2 μm, **(B)** represents 100 nm, **(F,H)** represent 200 nm.

After meiosis, tetrads of haploid microspores fill the anther locules (**Figure 1B**). Tetrads are surrounded by a thick asymmetric callose envelope and the primary cell wall is visible outside of the callose. The plasmalemma of the microspores is initially straight and in direct contact with the callose (**Figure 2A**). Subsequently, a fibrillar surface coat develops between the callose and the plasmalemma, which has a loose, irregular fibrillar texture and is considered primexine matrix (**Figure 2B**). Rod-shaped electron-dense units are radially oriented in the distal part of the primexine, which eventually form the columellae. At this stage, the epidermis, endothecium, and ML become high vacuolated, nuclei are displaced to the wall and nucleoli are fragmented. The ML becomes flat (**Figure 1B**). Tapetal cells contain numerous ribosomes and extensive layers of endoplasmic reticulum that lie below the cell membrane and surround the nuclei in concentric rings. Clusters of small vesicles are present throughout the tapetal cytoplasm and pre-Ubisch bodies are formed (**Figure 2C**).

At uninucleate stage, microspores are released from the tetrads into the anther locule, and increase both in volume and in wall thickness (**Figure 1C**). The epidermis and endothecium are high vacuolated and contain lipidic materials and starch grains, whereas the ML is almost degenerated. Abundant Ubisch bodies are produced in the tapetal cells and extruded through their inner tangential and radial walls (**Figure 2D**). The Ubisch bodies possess an irregular and granular shape. The electron density of the Ubisch body wall is very similar to that of the exine. During the early development of free microspores, the columellae increase in thickness, particularly at their distal ends, the tectum develops, while the sporopollenin-like materials are deposited between the distal portions of the columellae. The FL occurs at the bases of the columellae (**Figure 2E**). White-line-centered lamellae were observed between the FL and the endexine (**Figure 2F**). The endexine continues to develop on the proximal surface of these white lines and abundant electron-dense material, probably sporopollenin, appears beneath the endexine. Subsequently, the columellae become solid and the intercolumellar space is filled with fibrillar materials, i.e., remnants of the primexine matrix. As the microspores form a large vacuole (**Figure 2G**), the plasmalemma in the interapertural regions recedes, creating a large PS under the radially oriented membranous granular material layer (**Figure 2H**). This event might indicate the start of intine development. At this phase, the tapetal cell walls and cytomembranes are completely degenerated, a large number of vesicles and lipids are present throughout the tapetal cells and Ubisch bodies are released from the surface of the tapetal cells into the locule (**Figure 2I**). At this phase, a thin layer of cuticle is present at the anther epidermis (**Figure 2I**).

**FIGURE 3:**
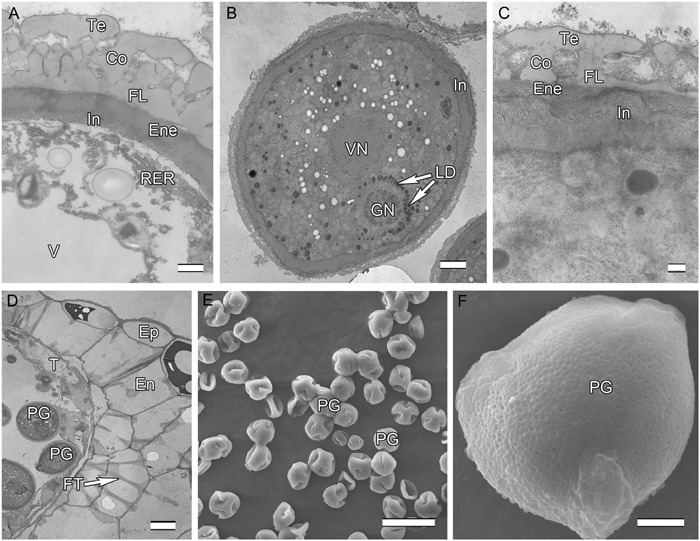
**Ultra-thin sections of *H. patens* anthers. (A)** Uninucleate microspore stage, showing the early-formed intine and associated RER. **(B)** Bicellular pollen stage, showing the GN surrounded by LD. **(C)** Bicellular pollen stage, showing the thickened and more electron-dense intine. **(D)** Bicellular pollen stage, showing cell layers of the anther wall, fibrous thickenings in the endothecium, and remnants of the tapetum. **(E)** MPGs released from the anther locule. **(F)** One PG, showing the micro-reticulate ornamentation and PO. Scale bars in **(A)** represents 500 nm; **(B)** represents 2 μm; **(C)** represents 200 nm; **(D)** represents 10 μm; **(E)** represents 50 μm; **(F)** represents 5 μm.

Initiation of intine development takes place prior to the first pollen mitosis. At this stage the intine has a fibrillar structure and microspores possess abundant organelles, e.g., RER and a large vacuole (**Figure 3A**). The cytoplasm contains long profiles of RER parallel with the plasmalemma and numerous mitochondria. Later the microspores enter BCP stage in which the generative cell is surrounded by LD (**Figure 3B**). The cytoplasm is characterized by an abundance of compound starch grains and LD (**Figure 3B**). The intine thickens and appears more electron-dense and reaches the endexine at several sites (**Figure 3C**). Initiation of the characteristic FT takes place in the endothecium (**Figure 1D**). Cells of the tapetum and ML completely degenerate, leaving the endothecium as the innermost anther wall layer (**Figure 3D**). The volume of the PG progressively increases, resulting in stretching of the pollen wall, which is more pronounced in PG at dehiscence. Projection of the intine in the aperture region was observed in some PG (**Figures 3D,F**). Pollen grains are bicellular at anthesis, relatively small [P (polar axis) 18.36 (17.8–19.4) μm × E (equatorial diameter) 19.66 (18.7–20.9) μm], and oblate spheroidal in equatorial view (**Figure 3E**). The grains are planaperturate with three compound apertures. The exine pattern is micro-reticulate with the sculpting somewhat obscured by the presence of fibrillar material that fills the spaces between the columellae (**Figure 3F**).

### cDNA Sequence Generation and *De novo* Assembly

Approximately 948 million bases were generated and a set of 105 million 90 bp paired-end reads were obtained after cleaning and quality checks. Q20 and Q30 values reached 97.8 and 92.1%, respectively, representing high quality sequencing. Assembly of reads resulted in 177,548 contigs with an average length of 354 nt. These were assembled into 89,849 unigenes, which were divided into two classes. The first class is distinct singletons (52,379) with the prefix unigene, representing unigenes derived from a single gene. The second class is distinct cluster unigenes with the prefix CL and the cluster ID, where each cluster contains unigenes with similarities of more than 70%. The 37,470 cluster unigenes are distributed into 14,948 distinct clusters, among which, 3,263 contain one unigene (unigenes shorter than 200 bp were removed after family clustering), and 7,247 contain two unigenes. The remaining 4,438 clusters contain three or more unigenes, and the largest two clusters contain 45 unigenes. The cluster unigenes may be derived from the same gene or from a homologous gene (Grabherr et al., 2011).

### Gene Annotation and Functional Classification

In the whole unigene set, a total of 57,476 (64%) unigenes were significantly matched to known genes in the public databases of NR, NT, Swiss-Prot, KEGG, COG, and GO (**Table 2**), representing putative functional annotations for more than half of the assembled sequences.

**Table 2:** Annotation results of *Hamelia patens* unigenes.

Sequence File	NR	NT	Swiss-Prot	KEGG	COG	GO	ALL
*H. patens-*Unigene	55,563 (61.8%)	46,601 (51.9%)	35,244 (39.2%)	31,169 (34.7%)	18,948 (21.1%)	42,244 (47.0%)	57,476 (64.0%)

Homology searches performed using BLASTx against the non-redundant (NR) protein database showed 55,563 (61.8%) of unigenes with matches to the NR database with an *E*-value cut-off of 1e-5 (Supplementary Figure S1A). The similarity distribution shows 68.6% of BLASTx hits are within the range 60–100%, representing strong homology. Only 31.4% have similarity values less than 60%, showing moderate homology (Supplementary Figure S1B). With regard to species similarity, the highest proportion of matched sequences in the NR database are derived from *Solanum lycopersicon* (33.9%, 18,815 unigenes) and *Vitis vinifera* (26.4%, 14,667 unigenes) which belong to the Solanales, a sister order to the Gentianales, to which Rubiaceae belongs (APG III, 2009; Supplementary Figure S1C). *H. patens* shows a similar proportion (27.2%, 15,090 unigenes) of sequences matching the genome of *Coffea canephora*, which was published recently (Denoeud et al., 2014; Dereeper et al., 2015). Further comparative analysis between *H. patens* and *C. canephora* is presented in section “Comparison with the Coffee Genome” below.

To understand the potential function of the assembled unigenes, the COGs (clusters of orthologous group of proteins) functional annotation system was used. In total, 18,948 (21.1%) unigenes were mapped to the COG database and possible functions and statistics were predicted. The five largest categories are general function (17.9%), transcription (8.9%), replication, recombination, and repair (8.6%), signal transduction mechanisms (7.0%), and posttranslational modification, protein turnover, and chaperones (6.9%) (Supplementary Figure S2). The proportion of genes annotated by COG and the predicted categories are quite similar in other studies of non-model species without known genomic sequences in the past a few years; for instance, 20.4% in *Triadica sebifera* in Euphorbiaceae (Divi et al., 2016), 20.1% in *Uncaria rhynchophylla* in Rubiaceae (Guo et al., 2014), and 21.1% in *Dendrocalamus latiflorus* in Gramineae (Zhang et al., 2012).

Gene ontology assignments were used to classify the functions of predicted genes, resulting in 42,244 (47.0%) unigenes assigned to at least one GO term, while 47,605 (53.0%) could not be assigned. Among the 42,244 BLASTable (*E*-value < 1e-5) unigenes, 17,923 unigenes are distinct singletons, while 24,321 are distinct cluster unigenes, distributed in 9,639 clusters. If the unigenes contained in the distinct clusters were treated as single unigenes, then the total number of annotated unigenes would be 27,562, and the annotated proportion would be 41.0%, which is close to the proportion of unigenes in the same cluster treated as independent unigenes. Non-BLASTable (*E*-value > 1e-5) sequences have been reported in all studied plant transcriptomes, with the proportion varying from 13.0 to 80.0%, depending on the species, the sequencing depth and the parameters of the BLAST search (Parchman et al., 2010; Blanca et al., 2011; Ness et al., 2011; Zhang et al., 2012, Zhang F.J. et al., 2014). Non-BLASTable (E-value > 1e-5) sequences might result from biological factors, including rapidly evolved genes with divergent sequences, species-specific genes, and the persistence of non-coding fractions derived mainly from untranslated regions (Logacheva et al., 2011; Zhang et al., 2012). The BLASTable (*E*-value ≤ 1e-5) unigenes are divided into three GO categories: biological process (32,700), cellular component (33,751), and molecular function (31,293). In the biological process category, the most abundant sequences are classified into cellular process (81.0%), metabolic process (77.0%), and single-organism process (58.4%). In the cellular component category, cell (93.6%), cell part (93.6%), and organelle (74.6%) are the three most represented GO terms. In the molecular function category, catalytic activity (66.7%), binding (63.6%), and transporter activity (10.0%) are highly represented (Supplementary Figure S3). The constitution of the most represented GO terms shows a high degree of similarity with the GO annotation of *C. canephora* (Dereeper et al., 2015).

To identify biological pathways activated during anther development of *H. patens*, the assembled unigenes were annotated against the KEGG database (*E*-value < 1e-5). A total of 31,169 (34.7%) unigenes were mapped into 128 pathways in the KEGG database (**Supplementary Table S2**). The maps with the highest unigene representation are metabolic pathways (6,975, 22.4%), followed by biosynthesis of secondary metabolites (3,465, 11.1%), plant-pathogen interaction (1,796, 5.8%), and plant hormone signal transduction (1,609, 5.2%). This constitution exhibits a high degree of similarity with rice anther transcriptome profiles derived from microarray analysis (Deveshwar et al., 2011).

### Comparison with the Coffee Genome

Alignments of the *H. patens* unigenes (89,849) with the coffee genome (25,574 CDSs) revealed 15,090 (16.8%) unigenes with a significant overlap to 7,313 (28.6%) coffee CDSs. This proportion is lower than expected given that both species are from the same family. When clean reads (105 million) were mapped to the coffee genome allowing a maximum 2 bp mismatch, a total of 366,227 (0.35%) reads matched 4,047 (15.8%) coffee CDSs, a lower proportion than observed for unigene alignments. Meanwhile, the number of reads with no mismatches was 8,821, corresponding to 286 coffee CDSs, and 79,220 reads with a 1 bp mismatch corresponding to 1,549 coffee CDSs. It is predictable that as mismatched bases increase, more matched reads and coffee CDS will result. Read alignment is presumed to be more accurate, since the reads represent the most comprehensive data. The low proportion of matches between *H. patens* and *C. canephora* might be explained by the fact that these species are not genetically close and are members of distant tribes in different subfamilies.

Among the 7,313 coffee CDSs, 3,774 have just one matching unigene, while the remaining 3,539 match multiple unigenes. In these cases, most of those matching unigenes are from the same cluster and are longer than the matched coffee CDS. This is indicative of sequence similarities other than matches with different portions of the same coffee CDS. Among these aligned unigenes of *H. patens*, 80 (0.5%) are related to anther wall and pollen wall development (**Supplementary Table S3**). The remaining unigenes are largely annotated with response to salt stress, DNA-dependent regulation of transcription, oxidation-reduction process, response to cadmium and protein phosphorylation, which represent general functions.

### Digital Gene Expression Tag Profiling

**FIGURE 4:**
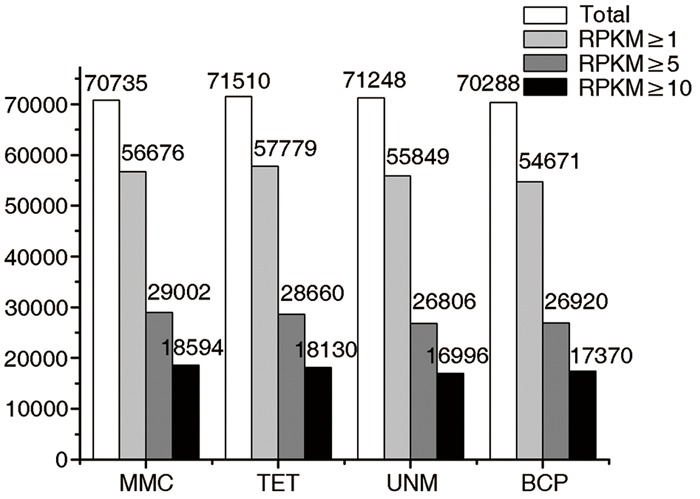
**Digital gene expression (DGE) libraries of four developmental stages.** The white bars in this bar graph depict the numbers of genes mapped by clean reads in each library. The light gray bars depict the numbers of genes with RPKM value ≥ 1 in each library. The dark gray bars depict the numbers of genes with RPKM value ≥ 5 in each library. The black bars depict the numbers of genes with RPKM value ≥ 10 in each library.

**FIGURE 5:**
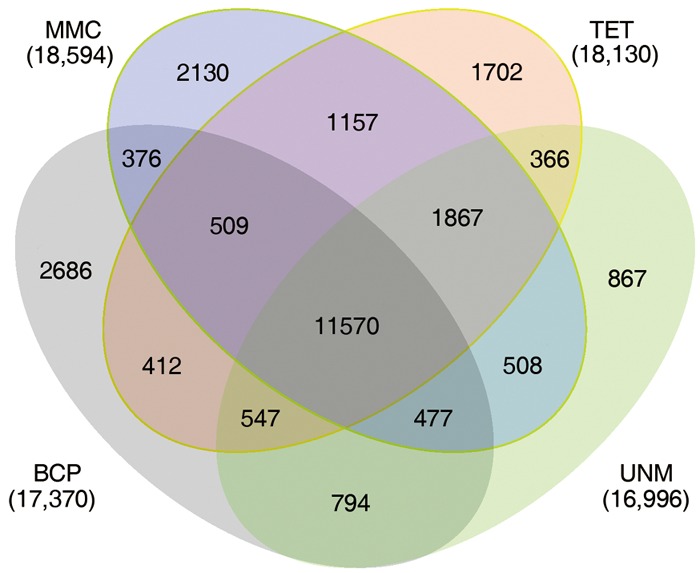
**Expression patterns of highly expressed genes.** The Venn diagram shows the constitution of genes highly expressed in at least one stage. The overlaps represent genes simultaneously highly expressed at two, three, or four stages.

The numbers of unigenes mapped by clean reads in each of the four libraries are 70,735 (MMC), 71,510 (TET), 71,248 (UNM), and 70,288 (BCP). Among these, 29,002 (41.0%), 28,660 (40.8%), 26,806 (37.6%), and 26,920 (38.3%) genes have RPKM values ≥ 5 in MMC, TET, UNM, and BCP, respectively. Detecting low abundance transcripts is one of the advantages of RNA-seq. In this study, 14,059 (19.9%), 13,731 (19.2%), 15,399 (21.6%), and 15,617 (22.2%) genes are low abundance in MMC, TET, UNM, and BCP, respectively, with RPKM ≤ 1. Genes with RPKM ≥ 10 at one or more stages were considered highly expressed and the numbers detected at MMC, TET, UNM, and BCP stages were 18,593 (26.3%), 18,129 (25.4%), 16,995 (23.9%), and 17,370 (24.7%), respectively, (**Figure 4**). It is supposed that unigenes with higher expression are more likely to be full-length, therefore further analyses was focused on genes with RPKM values ≥ 10 at one or more stages. These are classified into four main categories and 15 sub-categories based on expression pattern. The four main categories are highly expressed (RPKM value ≥ 10) in one, two, three, or in all four stages. The numbers of genes of each sub-category are calculated and illustrated in the Venn diagram (**Figure 5**). In total, 11,570 genes are highly expressed across all stages, which may suggest their involvement in housekeeping functions or general metabolism.

#### Identification of Up and Down-Regulated Genes and Their GO Annotations

**FIGURE 6:**
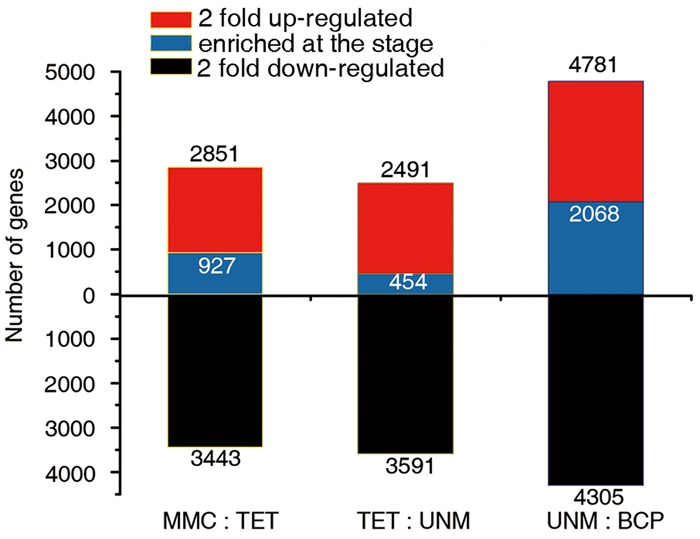
**Comparisons of adjacent developmental stages.** The DGE libraries of four stages were compared to their preceding stage during anther development. MMC has no reference. The number of genes twofold up-regulated or down-regulated at FDR ≤ 0.001 are plotted on the red or black bars in the graph. Among the up-regulated genes at each stage, the numbers of specifically enriched genes (RPKM value ≥ 10) are plotted on the blue bars.

The comparisons of adjacent stages, identified 2,851, 2,491, and 4,781 genes that were potentially up-regulated by at least twofold, while 3,443, 3,591, and 4,305 genes were potentially down-regulated in TET, UNM, and BCP stage, respectively. A greater number of genes were down-regulated in comparison to those up-regulated in TET and UNM, however, this trend is reversed in BCP where a larger proportion of genes showed up-regulation (**Figure 6**). Among these putatively up-regulated genes, those with high expression (RPKM value ≥ 10) in one particular stage and lower expression (RPKM < 10) in the other three stages, were considered stage-enriched. 927 (32.5%), 454 (18.2%), and 2,068 (43.3%) genes are enriched in TET, UNM, and BCP, respectively (**Figure 6**). BCP has the largest proportion of stage-enriched genes in all the stages analyzed, moreover, both up- and down-regulated genes increase dramatically in BCP in comparison to earlier stages, highlighting the distinct gene expression profile at this stage.

Functional association of the potentially up-regulated and stage-enriched genes based on GO annotations (**Supplementary Table S4**) revealed that TET stage-enriched and up-regulated genes were enriched in protein phosphorylation, metabolic process, oxidation-reduction process, histone H3-K9 methylation, DNA methylation, DNA-dependent regulation of transcription. UNM stage-enriched and up-regulated genes were enriched in oxidation-reduction process, DNA-dependent regulation of transcription, proteolysis, metabolic process, transmembrane transport and response to cadmium ion. Categorization of BCP stage-enriched and up-regulated genes into GO functional groups showed enrichment for genes involved in oxidation-reduction process, pollen tube growth, plant-type cell wall modification, protein phosphorylation, and transmembrane transport, which could be important contributors to pollen maturation and the pollen transcriptome. However, a large number (38,440) of BCP expressed genes have not been previously reported, which could serve as a useful resource to mine transcripts for validation of putative gametophyte and/or germline functions.

Genes with GO terms (**Supplementary Table S5**) related to anther wall and pollen wall development were further selected (Supplementary Figure S4). Among the putatively up-regulated genes in TET stage, 40 (46.5%) are related to pollen exine development, 40 (46.5%) are related to other components of, or contributing to, pollen wall development and six (7.0%) are related to tapetum development. Among the stage-enriched genes in TET stage, six (35.3%) are related to pollen exine development, nine (52.9%) to pollen wall development, and two (11.8%) to tapetum development. At the UNM stage, 11 (29.0%) putatively up-regulated genes are related to pollen exine development and 27 (71.0%) are related to other components of, or contributing to, pollen wall development, but none to tapetum development. In addition, there is one stage-enriched gene (Unigene6816) related to pollen exine development and one (Unigene9576) contributing to hemicellulose metabolic process, but none contributing to tapetum development. At the BCP stage, 50 (21.4%) putatively up-regulated genes are related to pollen exine development, 180 (76.9%) to other components of or contributing to pollen wall development and four (1.7 %) to tapetum development. At BCP stage, 20 (24.1%) stage-enriched genes are related to pollen exine development, 63 (75.9%) to other components of or contributing to pollen wall development and none related to tapetum development. From TET stage onward, the genes related to pollen wall development first decrease in UNM then increase in BCP, while the genes related to tapetum development decrease in both UNM and BCP. This trend is consistent with developmental events in pollen wall and tapetum.

#### Identification of Putatively Stage-Specific Expressed Genes and Their Expression Profiles

**FIGURE 7:**
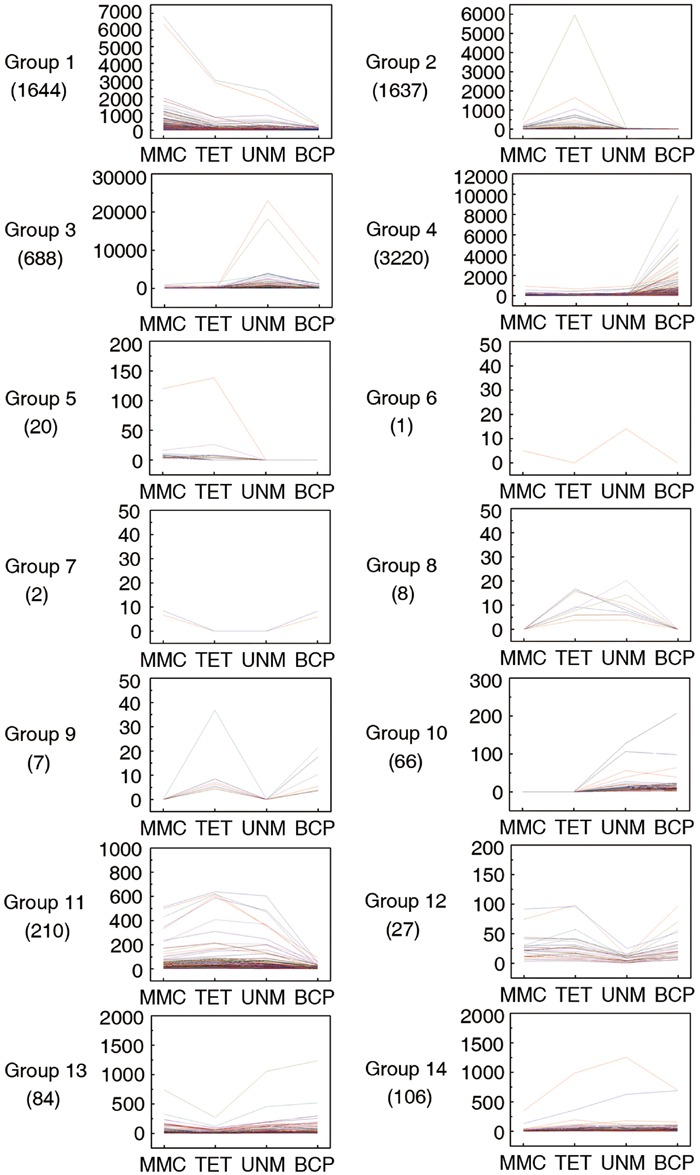
**Pairwise comparisons within four DGE libraries.** Genes with similar expression patterns were grouped together to make 14 groups. The line graphs show the variation of expression levels at four stages of anther development.

Pairwise comparisons within the four DGE libraries identified 7,720 genes are differentially expressed across the four stages. These genes were categorized into 14 groups based on up or down regulation. Hierarchical cluster analysis was performed on each of the groups to illustrate the results (**Figure 7**).

In group one to four, the genes are at least twofold up-regulated in one particular stage comparing to any of the other three stages and the differences in expression level are not significant. In total, 1,644 (21.3%), 1,637 (21.2%), 688 (8.9%), and 3,220 (41.7%) genes illustrated in the groups one to four show expression peaks in MMC, TET, UNM, and BCP, respectively. GO annotation analysis of the differentially expressed genes (**Supplementary Table S6**) indicated that a large number of genes were involved in oxidation-reduction processes, protein phosphorylation, pollen tube growth, response to salt stress, and response to cadmium ion. A total number of 86 genes (1.1%) in the groups 5 and 10 show twofold up-regulation in the former or the latter two stages, coinciding with the time of tapetum formation or tapetum PCD. These genes participate in oxidation-reduction processes, metabolic processes and DNA-dependent regulation of transcription. Genes in group 7 are down-regulated at tetrad and UNMs, while genes in group 8 are twofold up-regulated at these two stages. The genes in both groups were mainly enriched for protein ubiquitination. Genes in group 6 are down-regulated at tetrad and BCP stages, whereas group 9 genes are twofold up-regulated at these stages. Group 9 genes are involved in metabolic processes, sodium ion transmembrane transport, regulation of pH, pollen tube growth, and plant-type cell wall modification. In groups 11 to 14, a total of 427 (5.5%) genes are twofold down-regulated in one particular stage. The genes in these four groups were mainly related to oxidation-reduction process, ATP catabolic process, translation, DNA-dependent regulation of transcription. In total 3,427 (44.4%) genes in all 14 groups were not annotated by GO terms and as such, deserve further attention as a source of genes with unidentified roles in anthers.

**FIGURE 8:**
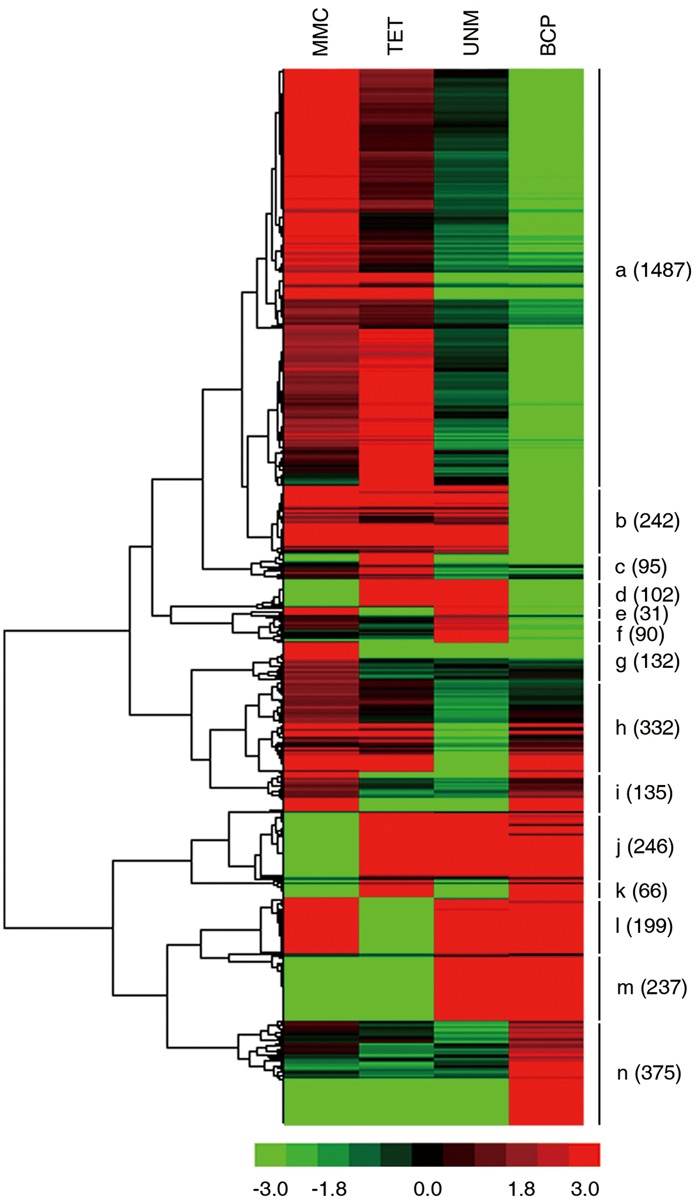
**Expression patterns of stage-specifically expressed genes.** Hierarchical cluster diagram represents expression patterns of stage-specifically expressed genes of four stages. The numbers of genes under each cluster are showed at the right side.

Groups one to four were further analyzed to identify stage-specifically enriched genes. They were defined as twofold up-regulated and highly expressed (RPKM ≥ 10) in one particular stage, while expression values in the other three stages were less than 10. As a result, 3,769 stage-specifically enriched genes across the four stages were obtained. The numbers at each stage were, 776 (20.6%), 807 (21.4%), 322 (8.5%), and 1,864 (49.5%) at MMC, TET, UNM, and BCP, respectively (**Figure 8**). UNM had the lowest share of stage-specifically enriched genes, while BCP had the largest share. Thus, BCP showed the most diverse expression profiles compared to the other three stages.

**FIGURE 9:**
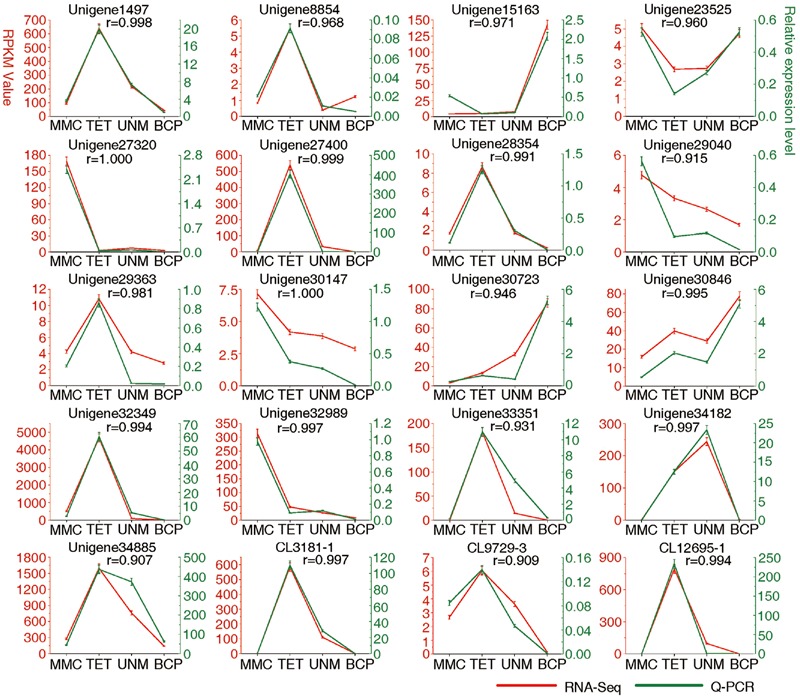
**Result of q-PCR analysis.** The left *Y* axis represents RPKM value of each gene using RNA-Seq analysis. The right *Y* axis represents log_2_ transformed relative transcript amount obtained by Q-PCR. The correlation co-efficient (r) between the two expression profiles is also showed.

The expression patterns of all stage SEGs were analyzed and genes with similar expression profiles were clustered. A total of 14 clusters were generated (**Figure 9**). Genes in the same cluster are considered co-expressed and may be targets of the same TFs. The expression profile of the largest cluster (“a”; 1,487 genes) showed up-regulation in MMC and TET followed by down-regulation in UNM and BCP, coinciding with the pattern of tapetum development. Further, GO terms relating to tapetum development are strongly enriched in cluster “a” (**Supplementary Table S7**) confirming the cytological changes of tapetum development from MMC to BCP involve gene down-regulation. Clusters “b” (242) and “g” (132) also exhibit down-regulation from MMC to BCP and are dominated by genes related to oxidation-reduction process, DNA-dependent regulation of transcription and metabolic process. Clusters “j” (246), “m” (237), and “n” (375), show up-regulation trends from MMC to BCP and are dominated by genes related to oxidation-reduction process, pollen tube growth, plant-type cell wall modification, and cellular membrane fusion. In clusters “d” (102), “c” (95), and “f” (90), genes were up-regulated at both or either of the TET and UNM stages and annotated by lipid metabolic process, very long-chain fatty acid metabolic process, transmembrane transport, and intracellular signal transduction. These features coincide with the finding that tapetal cells are most active during TET and UNM stages and are known to be involved in the synthesis of flavonoids and other secondary metabolites that eventually are transported to developing microspores. In clusters “i” (135), “l” (199), and “h” (332), genes are down-regulated at both or either of the TET and UNM stages and annotated by pollen tube growth, oxidation-reduction process, plant-type cell wall modification, and negative regulation of PCD. Genes in cluster “k” (66), showing up-regulation at TET and BCP stages, are mainly involved in xylan biosynthetic process, glucuronoxylan metabolic process, plant-type cell wall modification, cellulose biosynthetic process, and plant-type cell wall biogenesis. Coincidently, callose wall, primexine, and intine related to polysaccharide metabolism are synthesized mainly at TET and BCP stages. Genes down-regulated at TET and BCP stages are clustered in “e” (31), and are enriched in ATP catabolic process, metabolic process, transmembrane transport, and DNA-dependent regulation of transcription.

#### Candidate Genes Relevant to Tapetum and Pollen Wall Development

Differentially expressed genes and SEGs enriched for the relevant GO terms of anther wall and pollen wall development were identified. In total 243 differentially expressed genes and 108 stage-specific genes were obtained (Supplementary Figure S5). Genes related to sporopollenin biosynthetic processes are only enriched at TET (6) and UNM (1) stages. Genes related to pollen exine formation, are mainly enriched at TET (26 genes) and BCP (35 genes) stages, among which 5 (19.2%) and 19 (54.3%) genes are specifically expressed. Only one gene related to tapetal layer morphogenesis is specifically expressed at TET stage. There are four and three genes related to tapetal layer development differentially expressed at MMC and TET stages, respectively. Among those genes, three are specifically expressed at MMC and one is specifically expressed at TET stage. Genes relating to pectin are mainly enriched at MMC and BCP stages, with 14 (MMC) and 26 (BCP) genes being differentially expressed and 11 of which are specifically expressed at BCP stage. Genes relating to cellulose mainly express at MMC, TET, and BCP stages, with the number of differentially expressed genes being 15, 12, and 41, and the number of SEGs as 5, 4, and 20, respectively. Genes corresponding to hemicellulose metabolic processes are only expressed at TET, UNM, and BCP stages, whilst 88.4% (38 genes) of these are differentially expressed at BCP stage and among these genes, 30 are specifically expressed. These findings are consistent with the cytological changes and developmental events in anther wall and pollen wall development.

Pollen wall development requires lipid and polysaccharide metabolism, therefore genes enriched in carbohydrate and lipid metabolism pathways were analyzed (Supplementary Figure S6). The numbers of differentially expressed genes in both pathways only slightly change between MMC and UNM stages, while a marked increase occurs at BCP stage. This trend emphasizes the distinct gene expression profile at BCP stage. At MMC and UNM stages, a greater number of stage-enriched genes are involved in lipid metabolism in comparison to those involved in carbohydrate pathways (**Supplementary Table S8**). At TET and BCP stages, this trend is reversed, where fewer stage-enriched genes are involved in lipid metabolism than in carbohydrate metabolism. The MMC stage is enriched with a greater number of genes relating to glycerophospholipid and lipid metabolism, while TET stage is enriched in starch and sucrose metabolism as well as glycerophospholipid metabolism. UNM is enriched with the greatest number of genes relating to cutin, suberin, and wax biosynthesis. The first three largest numbers of genes at BCP are related to starch and sucrose metabolism, pentose and glucuronate interconversions, and glycerophospholipid metabolism. This might suggest nutrient synthesis and storage at BCP stage, consistent with the observation that the BCP cytoplasm is characterized by an abundance of starch grains and LD.

### Verification of Expression Profiles of Selected Unigenes Related to Tapetum and Pollen Wall Development by qRT-PCR

Real-time quantitative PCR (qRT-PCR) analysis was carried out on 20 candidate genes selected at random (**Figure 9**). Among these, 14 genes show high expression with RPKM ≥ 10 in at least one stage, while six display low expression with RPKM ≤ 10 at all four stages. The expression values at developmental stages derived from RNA-seq and q-PCR of each gene are listed in **Supplementary Table S9**. The expression patterns of all 20 genes analyzed by qRT-PCR largely agree with the DGE tag profiling, as the correlation co-efficients (r) are all greater than 0.9. This result provides support for the reliability of RNA-Seq data and the differential gene expression profiles observed for anther stages of *H. patens*.

## Discussion

### Tapetum and Pollen Development Display Common Features in Rubiaceae

Anther development is a complex process which involves transition from sporophyte to gametophyte, control of mitotic and meiotic cell divisions, together with the coordination of pollen and anther maturation (McCormick, 1993; Scott et al., 2004; Zhang et al., 2011). Ultrastructural changes involved in anther development have been described for many angiosperms, especially for model species including *Arabidopsis* and rice (Sanders et al., 1999; Zhang and Wilson, 2009; Zhang et al., 2011), although there are differences in the resolution and detail of the stages described (Owen and Makaroff, 1995). In our study, anther and pollen development of *H. patens* was shown to be typical of many angiosperms, with some features common in the Rubiaceae family. Our observations of developmental events and cytological changes provide a solid foundation for the analysis of gene expression profiles in the developing anthers of *H. patens*.

The tapetum cells in *H. patens* maintain integrity and position indicating that the tapetum is of the secretory type, which fits the description of a type five tapetum (Pacini, 1997), the most common in angiosperms (Owen and Makaroff, 1995). Ubisch bodies occurred simultaneously with the developing pollen exine, which corroborates the idea that they are required for transferring tapetum-derived sporopollenin precursors to the exine (Huysmans et al., 1998). The tapeta of *Arabidopsis* and rice are also secretory but use different export routes for sporopollenin. In *Arabidopsis*, specialized organelles, elaioplasts, and tapetosomes, are supposed to be transporters. Lipids are transported from the tapetum to microspores in two ways: vesicular transport and use of transporters as lipid carriers (Vizcay-Barrena and Wilson, 2006). In rice and other cereals, Ubisch bodies are thought to export sporopollenin across the hydrophilic cell wall to the locule (Zhang et al., 2011). Thus, the tapetum in dicots and monocots might have different mechanisms for sporopollenin translocation. Analogies are often found between the ornamentation of the pollen exine and that of the Ubisch body wall (Hesse, 1986; Vinckier and Smets, 2002, 2003). Ubisch bodies are considered have great potential as model system to study sporopollenin deposition, since they are acellular structures, independent of cytoplasmic control (Clément and Audran, 1993; Verstraete et al., 2014). To date, only the *RAFTIN* gene was identified in pro-orbicule bodies and shown to accumulate in Ubisch bodies in wheat and rice. *RAFTIN* is highly anther-specific and essential for pollen development in cereals (Wang et al., 2003). Future genetic studies to identify the genes and gene products that control sporopollenin polymerisation and exine patterning will need to take Ubisch bodies into account when screening for phenotypes (Verstraete et al., 2014).

The fundamental structure of pollen wall in angiosperms consisting of the outer exine and the inner intine is generally conserved (Heslop-Harrison, 1968; Blackmore et al., 2007; Ariizumi and Toriyama, 2011). The components contributing to the pollen wall are produced and accumulated in a precise temporal sequence. Sporopollenin makes up the majority of the material of the exine (Blackmore et al., 2007). In *H. patens*, the sporopollenin deposition starts from TET stage. Intine development starts from the late UNM stage and is complete by the end of BCP stage. The ontogenetic sequence of pollen wall development follows the basic scheme in the family (Hansson and El-Ghazaly, 2000; El-Ghazaly et al., 2001). Intinous projections occurred in the aperture region of *H. patens* PG, known as protruding onci or pollen buds in the Rubiaceae, was believed to be a relatively common feature in this family (Dessein et al., 2005; Kuang et al., 2012).

Besides exine and intine, the callose wall and primexine are successively synthesized and degraded at precise times. The callose wall, consisting of linear β-1,3-glucan polymers (Hird et al., 1993), begins to deposit at the beginning of TET stage and degrades at the end of TET stage in *H. patens*. The primexine, which acts as a template that guides the accumulation of sporopollenin, is composed of neutral and acidic polysaccharides, cellulose, and some proteins (Heslop-Harrison, 1968; Scott et al., 2004; Jiang et al., 2013). Primexine matrix usually with fibrillary texture appears between the callose and the plasmalemma at the TET stage in *H. patens.* In addition, lipidic material, which may be classed as pollenkitt (El-Ghazaly et al., 2001; Edlund et al., 2004), is deposited onto the exine surface at the late BCP stage. These ontogenetical features were also described in the available ultrastructural studies in Rubiaceae (Hansson and El-Ghazaly, 2000; El-Ghazaly et al., 2001; Kuang and Liao, 2015).

### DEGs Expression Patterns Agree with the Developmental Events

Genes related to sporopollenin biosynthetic process were closely associated with fatty acid metabolism and only expressed at TET and UNM stages, consistent with the appearance of pro-Ubisch bodies and Ubisch bodies in tapetal cells. Genes related to pollen exine formation are enriched at TET and BCP stages, which may correspond to the time that primexine formed and exine deposited. Genes related to hemicellulose metabolic process, are mainly enriched at BCP stage, a few are enriched at TET and UNM stages and none are expressed at MMC stage, coinciding with the stages at which the primary cell wall and intine develop. Genes related to pectin are largely enriched at BCP stage, and genes related to cellulose are largely enriched at TET and BCP stages. These gene expression patterns agree well with the observed stages in which intine and primexine appear in *H. patens*. Genes related to tapetal layer morphogenesis and development are enriched at MMC and TET stages, consistent with stages during which tapetal cells are differentiated and pro-Ubisch bodies are produced.

The numbers of genes enriched in different pathways are correlated with structures and components present at different anther developmental stages. At UNM stage, abundant Ubisch bodies are synthesized, which involve lipid metabolism. Consistently, more genes are revealed in lipid metabolism pathways than in carbohydrate metabolism pathways at the same stage. The synthesis and degradation of callose wall and primexine, and the formation of intine are involved in carbohydrate metabolic pathways (Jiang et al., 2013). The genes related to the carbohydrate metabolism are enriched at the stages during which callose wall, primexine, and intine form. In addition, the cytoplasm of mature *H. patens* PG is characterized by an abundance of compound starch grains and LD at BCP stage. This may account for the increase in the number of genes enriched in both carbohydrate and lipid metabolism pathways at this stage.

Gene ontology enrichment analysis of the stage-enriched genes and differentially expressed genes suggested the gene number, gene function, and expression profile were significantly correlated with anther developmental processes in *H. patens*. The genes filtered from KEGG pathways analysis and GO enrichment analysis, provide a useful resource to identify novel genes and their possible functions in pollen wall development.

### Comparative Analysis of Anther and Pollen Transcriptomes Confirms Inter-Specific Conserved Patterns

Developmental transcriptomic analysis of anthers and PG utilizing microarray platforms have been reported in *Arabidopsis* (Honys and Twell, 2004; Pina et al., 2005), rice (Huang et al., 2009; Fujita et al., 2010; Deveshwar et al., 2011), maize (Ma et al., 2008), and *Brassica rapa* (Dong et al., 2013). Our study revealed 89,849 unigenes in the *H. patens* anther transcriptome, well above the numbers of genes predicted to be expressed in anthers of other species. However, high-throughput sequencing techniques increase the chance of detecting transcripts with low abundance and microarrays have been reported to be less effective at identifying low abundance transcripts (Loraine et al., 2013). Further, the number of *H. patens* unigenes is an estimate, since the genome is unknown and unigenes sharing sequence similarities were classified as different unigenes, in consideration of alternative splicing events. High numbers of unigenes have also been reported for species with no reference genomes such as for the fruit transcriptome of *Triadica sebifera* with 92,550 unigenes (Divi et al., 2016), the embryo transcriptome of chrysanthemum with 116,697 unigenes (Zhang F.J. et al., 2014) and the leaf transcriptome of *Lycium chinense* with 61,595 unigenes (Wang et al., 2015). Finally, each developmental stage of *H. patens* anther contains about 20% of transcripts with RPKM ≤ 1, which most likely would not be detected with microarrays. These low abundance transcripts provide a resource for future analyses.

Transcriptomic analysis in developing and germinated pollen of rice displayed a “U-type” change in the number with the lowest number at the BCP stage (Wei et al., 2010). Developmental analysis of the *Arabidopsis* pollen transcriptome revealed a major shift in mRNA populations between BCP and TCP stages, reflective of the transition from earlier cell division to later pollen maturity (Honys and Twell, 2004). This distinct phase shift suggests inter-specific conservation of pollen-expressed genes. In *H. patens* anthers, BCP stage has the greatest number of stage-specific genes. The divergence between *H. patens* and the two model species might result from the relative contribution of sporophytic and gametophytic cell types and earlier developmental stages (MMC and TET) in *H. patens* together with taxon-specific features, such as insect-pollination and BCP at maturity. Moreover, different platforms, tissue collections and comparison methods can result in significant variation in anther transcriptome profiles for different species (Hollender et al., 2014).

**FIGURE 10:**
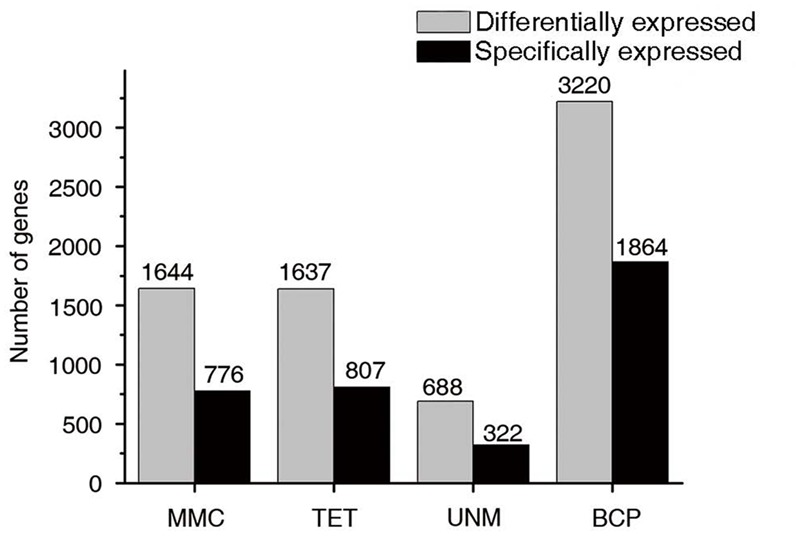
**Stage-specifically expressed genes at four developmental stages.** The gray bars in this bar graph depict the numbers of differentially expressed genes (genes in groups one to four in **Figure 7**). The black bars depict the numbers of specifically expressed genes (twofold up-regulated and RPKM value ≥ 10) in each library.

Since sample staging of anther development is similar between *H. patens* and rice in the study of Deveshwar et al. (2011), additional comparative analysis was carried out. Transcriptome profiling of rice was investigated in anthers at pre-meiotic (PMA), meiotic (MA), single-celled microspore (SCP), and tri-nucleate pollen (TPA) stages (Deveshwar et al., 2011). At PMA and MA stages (MMC in *H. patens*), there was high representation of sporophytic tissue compared to gametophytic tissue. This characteristic coincided with the phenomenon that most of the transcriptome changes corresponding to the sporogenous tissue and developing tapetum. TPA anthers contained a relatively higher cellular mass of gametophytic tissue (pollen), meanwhile prominent differences were found in transcript composition comparing with transcriptomes of earlier anther stages. In *H. patens*, BCP stage anthers were the most distinctive of the four stages, with the highest number of stage-specific and differentially expressed transcripts (**Figure 10**), showing great similarity to the TPA stage in rice (Deveshwar et al., 2011). This common shift, from free microspore stage anthers to late stage anthers, could be explained by the distinctive transcriptomes of developing pollen and sperm. It also reflects the large number of BCP enriched genes in *H. patens* associated with pollen development and maturation. Comparison of gene expression between two adjacent stages of anther development further highlighted this major switch of gene expression. The apparent transition from the free microspore stage to the BCP stage in *H. patens* also reflected the switch from the sporophytic to the gametophytic program (Deveshwar et al., 2011).

### Homologs of Anther and Pollen-Expressed Transcription Factors Were Revealed

Putative homologs of known TFs expressed in developing anthers were identified in *H. patens* (**Supplementary Table S10**). The expression patterns of these genes were largely consistent with those of their counterparts in other species, supporting the conservation of regulatory features in anther and pollen development in *H. patens*.

In the early stages of *Arabidopsis* anther development, DYT1 is required for tapetum development and function (Zhang et al., 2006), directly regulating *TDF1* expression in the tapetum (Feng et al., 2012). The highest expression of both *DYT1* and *TDF1* occurred at similar stages of tapetal development (Gu et al., 2014). CL1502.contig1 of *Hamelia patens* shares high amino acid sequence identity with *DYT1* in the bHLH domain. Unigene29472 is a putative homolog of *TDF1*. Both CL1502.contig1 and Unigene29472 show expression peaks at MMC stage, weak expression at TET or/and UNM stages, but no expression at BCP stage. *AtMYB103*, another important TF for tapetum function and male fertility, is also regulated by DYT1 (Feng et al., 2012). A candidate homolog of *AtMYB103*, CL6214.contig1, shows peak expression at MMC stage and lower expression at TET stage, but no expression at UNM and BCP stages. The expression pattern of these three putative homologs correspond to the early stages of tapetum development and callose dissolution, and exhibit somewhat consistent patterns with those of *DYT1, AtMYB103*, and *TDF1*, which suggests shared pathways in the regulatory network controlled by DYT1 in *Arabidopsis* and *H. patens*.

CL4992.contig3 shares high amino acid sequence identity with the MYB domains of *AtMYB97, AtMYB101*, and *AtMYB120.* It shows expression peak at BCP stage, indicating a consistent profile with these three MYB genes. DUO POLLEN1 (DUO1) is an *Arabidopsis* male germline-specific R2R3-type MYB TF (Durbarry et al., 2005; Rotman et al., 2005) that is essential for male germ cell division and differentiation (Brownfield et al., 2009). CL1108.Contig1, homologous to *AtDUO1*, shows peak expression at BCP stage, consistent with that of *AtDUO1*. It is likely that some of the pathways regulated by MYB factors are shared by *Arabidopsis* and *H. patens*.

The AtMIKC^∗^ TFs AGAMOUS-LIKE30 (AGL30), AGL65, AGL66, AGL94, and AGL104 are mainly expressed in mature pollen and play an essential role in transcriptional regulation during late pollen development (Verelst et al., 2007a,b). In *H. patens*, Unigene32598 and Unigene1629 share high amino acid sequence identity in the MIKC^∗^ domain, representing probable homologs of MIKC^∗^ TFs. Both of these are predominantly expressed at BCP stage, consistent with the expression patterns of AtMIKC^∗^ genes, which indicates conserved regulatory features of late pollen development and pollen maturation in *H. patens*.

### Expression Profiles of Homologs with Verified Conserved Patterns in Pollen Development

The *H. patens* homologs of known genes with characterized functions in exine formation in *Arabidopsis* or rice were identified (**Supplementary Table S10**). Their expression profiles were analyzed and verified by Q-PCR. *TKPR1*, which is involved in a conserved biosynthetic pathway in sporopollenin monomer biosynthesis, shows peak expression at tetrad stage (Grienenberger et al., 2010). Unigene32349, a homolog of *TKPR1*, is putatively highly expressed at TET stage, consistent with the expression profile of *TKPR1*. *LAP6* and *LAP5* are specifically expressed during the period of exine synthesis and are essential for the exine production (Dobritsa et al., 2010). The homologous unigenes CL12695Contig1 and CL3181Contig1 show peak expression at TET and UNM stages. *LAP3* is essential for pollen development and proper exine formation (Dobritsa et al., 2009), and the homologous Unigene27400 shows peak expression at TET stage. Unigene34182 shows peak expression at TET and UNM stages and represents the *H. patens* homolog of *MS2*, which is involved in exine development and is expressed in tapetum shortly after microspore release (Aarts et al., 1997; Gómez et al., 2015). Unigene33351 and CL9729-Contig3 show higher read counts at TET stage and are homologous to the gene *ABCG26/WBC27*. *ABCG26* is expressed specifically in tapetal cells at the early vacuolate stage and plays a crucial role in the transfer of sporopollenin lipid precursors from tapetal cells to anther locules (Choi et al., 2011), while *WBC27* is expressed during early stages of anther development (Xu et al., 2010).

Unigenes homologous to known genes related to tapetum development, intine development and callose synthesis were also identified in *H. patens* (**Supplementary Table S10**). Unigene30846, homologous to *AtUSP* (Schnurr et al., 2006), is suggested to be involved in intine development and putatively highly expressed at BCP stage, consistent with the period of intine accumulation. Moreover, Unigene30846 matches Cc10_g13640 of coffee and shares the functional GO annotation of UDP-sugar pyrophosphorylase, which suggests functional similarity. Unigene29363 is homologous to *CalS5*, which encodes a callose synthase (Dong et al., 2005). The annotation of Unigene29363 (0052543//callose deposition in cell wall, 0006075//(1->3)-beta-D-glucan biosynthetic process and 0009556//microsporogenesis) and peak expression at TET stage are consistent with the function and expression pattern of *CalS5. RTS2*, a unique gene in the rice genome, is required for tapetal development and is predominantly expressed during meiosis (Luo et al., 2006). Unigene8854 is homologous to *RTS2* and shows peak expression at TET stage corresponding to that of *RTS2*.

Collectively, the predicted functions and verified expression patterns of the aforementioned unigenes are consistent with those of their homologs in *Arabidopsis* and/or in rice. This phylogenetic conservation of gene expression further validates our analysis of the *H. patens* anther transcriptome.

### Potential Genes for Tapetum and Pollen Wall Development Provide Useful Resources for Future Study

Currently most characterized genes involved in tapetum or pollen wall development were identified based on genetic analysis of male sterile or reduced fertility mutants, including those with defective pollen wall development. Transcriptomic profiling is expected to expand knowledge of the genes involved in these developmental processes. In particular, our analysis of the *H. patens* anther transcriptome has allowed the identification of many more candidate genes involved in pollen development. We identified 243 differentially expressed genes and 108 stage-specific genes potentially related to tapetum layer morphogenesis and development, sporopollenin biosynthesis, exine formation, cellulose and pectin metabolism and biosynthesis, and hemicellulose and cellulose metabolism. The most significant alignments among these classes of genes derive from *Solanum lycopersicon, Vitis vinifera*, and *Coffea canephora*. Moreover, some potential orthologs in *C. canephora* have similar annotations relevant to pollen development, for instance, callose synthase and cellulose synthase. Low abundance transcripts expressed during anther development were also mined and differential expression patterns of genes in anthers and pollen were uncovered. This wealth of information lays the foundation for higher resolution genome-wide transcriptomic profiling of *H. patens*, functional investigation of the identified candidate genes, and the evo-devo exploration of angiosperm pollen.

## Data Accessibility

Clean Illumina reads: NCBI SRA: SRP079620.

## Author Contributions

YK conceptualized and designed this study. LY and YK performed the experiments. LY, YK, XZ, and DT performed data analysis. LY, YK, DT, and JL wrote the manuscript. All authors have read and approved the final version of the manuscript.

## Conflict of Interest Statement

The authors declare that the research was conducted in the absence of any commercial or financial relationships that could be construed as a potential conflict of interest.

## Footnotes

1 http://trinityrnaseq.sourceforge.net/

2 http://coffee-genome.org/download

3 http://www.geneontology.org/

4 http://www.yeastgenome.org/cgi-bin/web-primer

## Supplementary Material

TABLE S1 **Primers used in q-PCR**.

TABLE S2 **Result of KEGG annotation**.

TABLE S3 **Alignment result of the *H. patens* unigenes with the coffee genome**.

TABLE S4 **GO annotation of up-regulated and stage-enriched genes**.

TABLE S5 **GO terms selected for GO functional enrichment analysis**.

TABLE S6 **GO annotation of differentially expressed genes**.

TABLE S7 **GO annotation of clusters a–n**.

TABLE S8 **Numbers of genes enriched in carbohydrate and lipid metabolism pathways**.

TABLE S9 **Expression values from RNA-seq and q-PCR of each gene**.

TABLE S10 **Putative homologs of known TFs expressed in *H. patens* anthers**.

## Abbreviations

BCP: bicellular pollen
Ca: callose
Co: columellae
Ep: epidermis
Ec: endothecium
Ene: endexine
FL: foot layer
FT: fibrous thickenings
GN: generative nucleus
GPG: germinated pollen grain
In: intine
LD: lipid droplets
ML: middle layer
MMC: microspore mother cell
MPG: mature pollen grain
Ms: microspores
MSA: mixed stage anther transcriptome library
N: nucleus
Nu: nucleolus
PG: pollen grains
PO: protruding oncus
PrE: primexine
PS: pericytoplasmic space
RER: rough endoplasmic reticulum
RPKM: reads per kilo bases per million mapped reads
T: tapetum
TCP: tricellular pollen
TET: tetrad
Te: tectum
Tet: tetrads
UNM: uninucleate microspore
V: vacuole
VN: vegetative nucleus
