# Corrigendum: Transcriptome Analysis of *Hamelia patens* (Rubiaceae) Anthers Reveals Candidate Genes for Tapetum and Pollen Wall Development

**DOI:** 10.3389/fpls.2017.00369

**Published:** 2017-03-17

**Authors:** Lin Yue, David Twell, Yanfeng Kuang, Jingping Liao, Xianqiang Zhou

**Affiliations:** ^1^Key Laboratory of Plant Resources Conservation and Sustainable Utilization, South China Botanical Garden, Chinese Academy of SciencesGuangzhou, China; ^2^College of Life Sciences, University of Chinese Academy of SciencesBeijing, China; ^3^Department of Genetics, University of LeicesterLeicester, UK; ^4^Beijing Genomic InstituteShenzhen, China

**Keywords:** transcriptome, Rubiaceae, *Hamelia patens*, anther, tapetum, pollen wall

There was a mistake in the order of “Figure 8,” “Figure 9,” and “Figure 10” as published. “Figure 8” should be modified to “Figure 9.” “Figure 9” should be modified to “Figure 10.” “Figure 10” should be modified to “Figure 8.”

On page 13, the sentence “Real-time quantitative PCR (qRT-PCR) analysis was carried out on 20 candidate genes selected at random (Figure 9)” should be modified to “Real-time quantitative PCR (qRT-PCR) analysis was carried out on 20 candidate genes selected at random (Figure 10).”

On page 15, the sentence “In *H. patens*, BCP stage anthers were the most distinctive of the four stages, with the highest number of stage-specific and differentially expressed transcripts (Figure 10), showing great similarity to the TPA stage in rice (Deveshwar et al., 2011)” should be modified to “In *H. patens*, BCP stage anthers were the most distinctive of the four stages, with the highest number of stage-specific and differentially expressed transcripts (Figure 8), showing great similarity to the TPA stage in rice (Deveshwar et al., 2011).”

The authors apologize for the mistake. This error does not change the scientific conclusions of the article in any way.

## Conflict of interest statement

The authors declare that the research was conducted in the absence of any commercial or financial relationships that could be construed as a potential conflict of interest.

