# Supplementary material for: Transcriptome Analysis of Hamelia patens (Rubiaceae) Anthers Reveals Candidate Genes for Tapetum and Pollen Wall Development
Source: Front Plant Sci. 2017 Jan 6;7:1991. doi: 10.3389/fpls.2016.01991 (PMC5220384; doi:10.3389/fpls.2016.01991)
Supplement: Supplementary file 11 [file Data_Sheet_1.pdf]

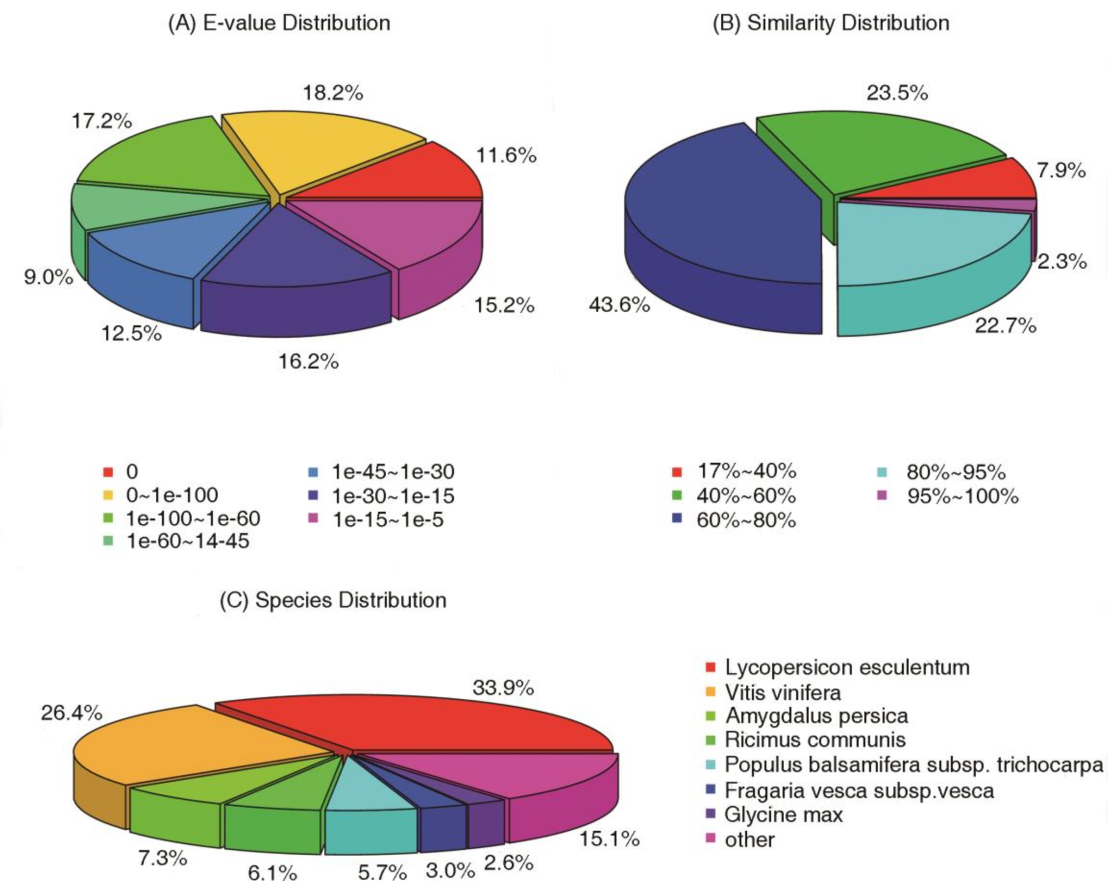

Supplementary Fig. S1 Result of NR Annotation.

(A) The E-value distribution of the result of NR annotation. (B) The similarity distribution of the result of NR annotation. (C) The species distribution of the result of NR annotation.

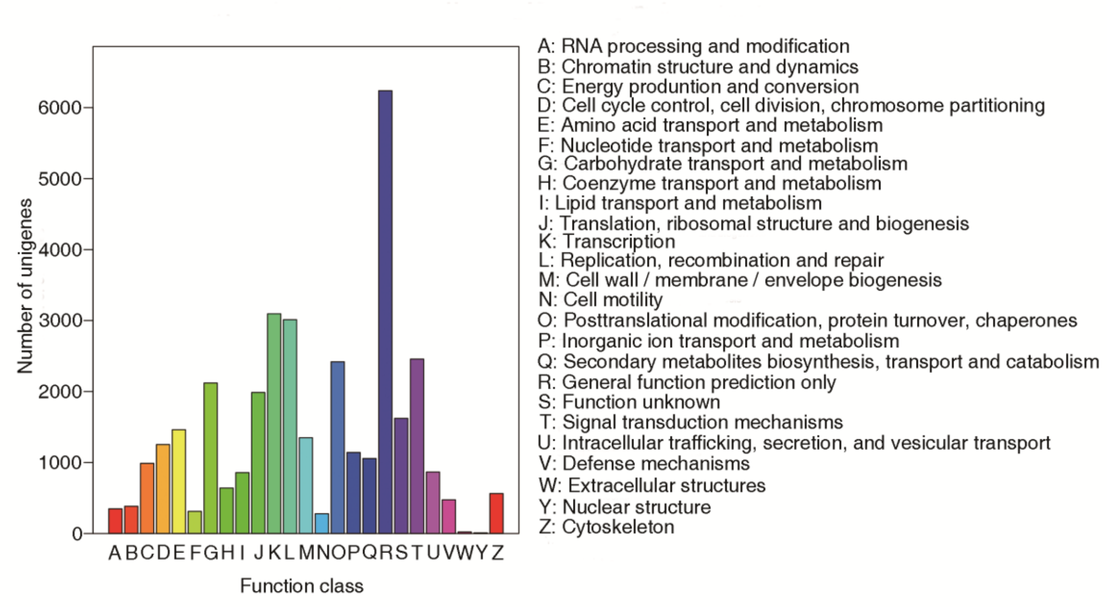

Supplementary Fig. S2 COG Function classification of *H. patens* unigenes.

The horizontal coordinates are function classes of COG and the vertical coordinates the numbers of unigenes in each class. The full names of the function classes are listed to the right.

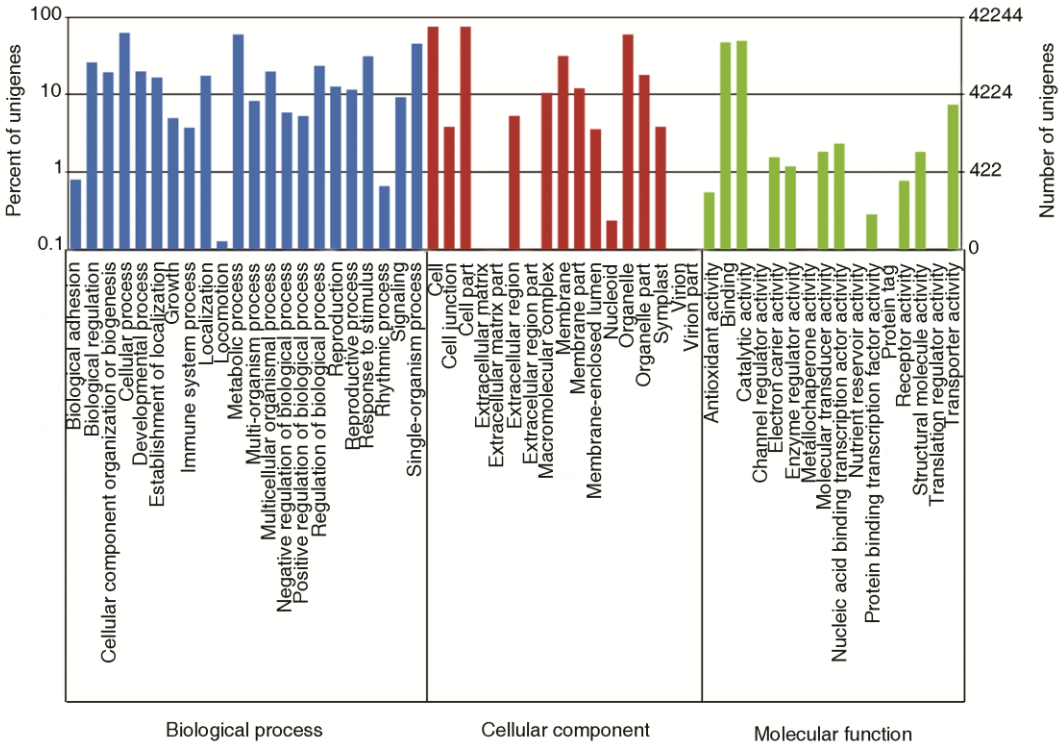

Supplementary Fig. S3 GO Classification of *H. patens* unigenes.

The X-axis shows the GO function classes. The right Y-axis shows the numbers of unigenes in one class and the left Y-axis the percentages of unigenes in each class.

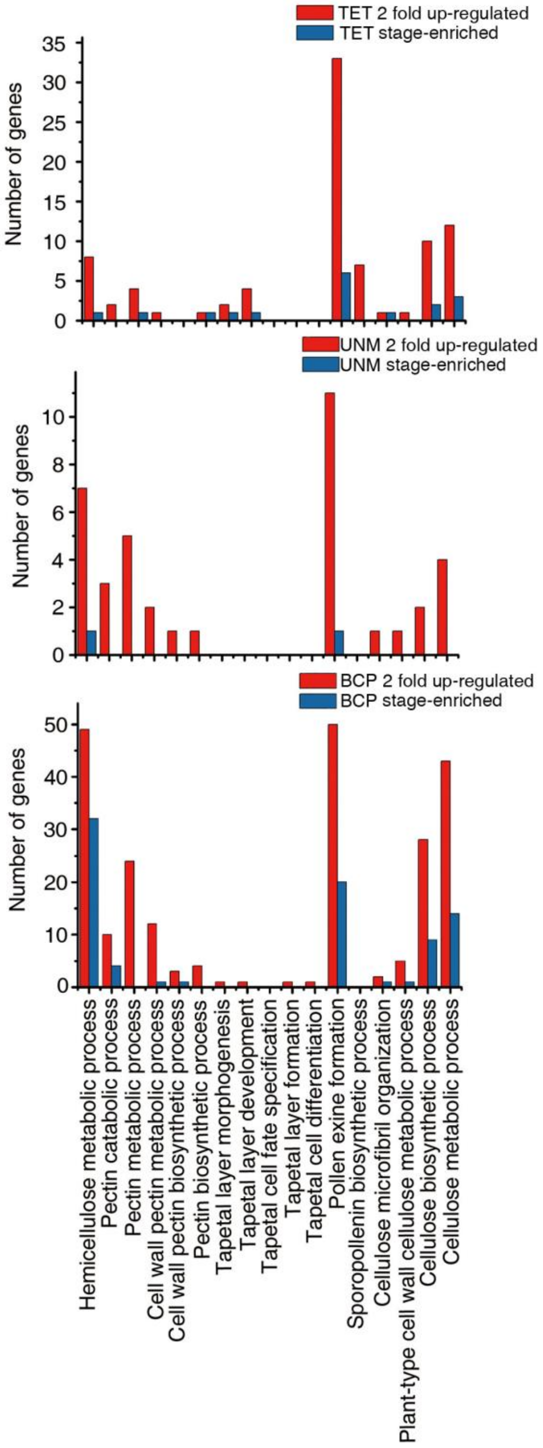

Supplementary Fig. S4 GO enrichment analysis of 2-fold up-regulated and specifically-enriched genes.

The bar graph highlighting the distribution of the up-regulated and enriched genes at individual stages into functional categories based on GO annotations.

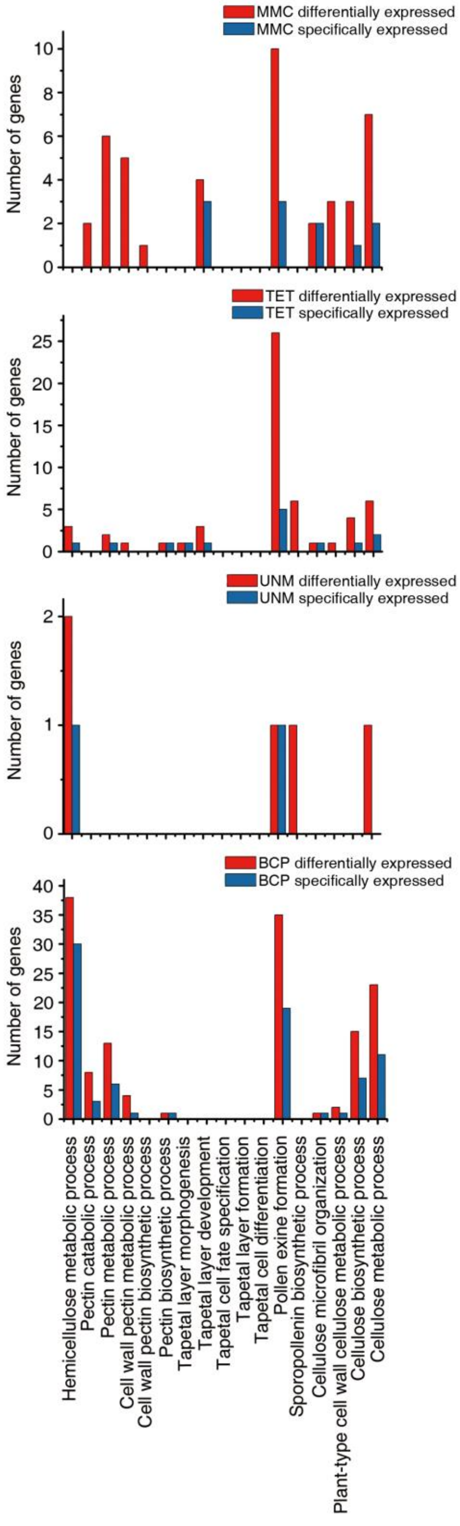

Supplementary Fig. S5 GO enrichment analysis of differentially expressed and stage-specifically expressed genes.

The bar graph highlighting the distribution of the differentially expressed and stage-specifically expressed genes at individual stages into functional categories based on GO annotations.

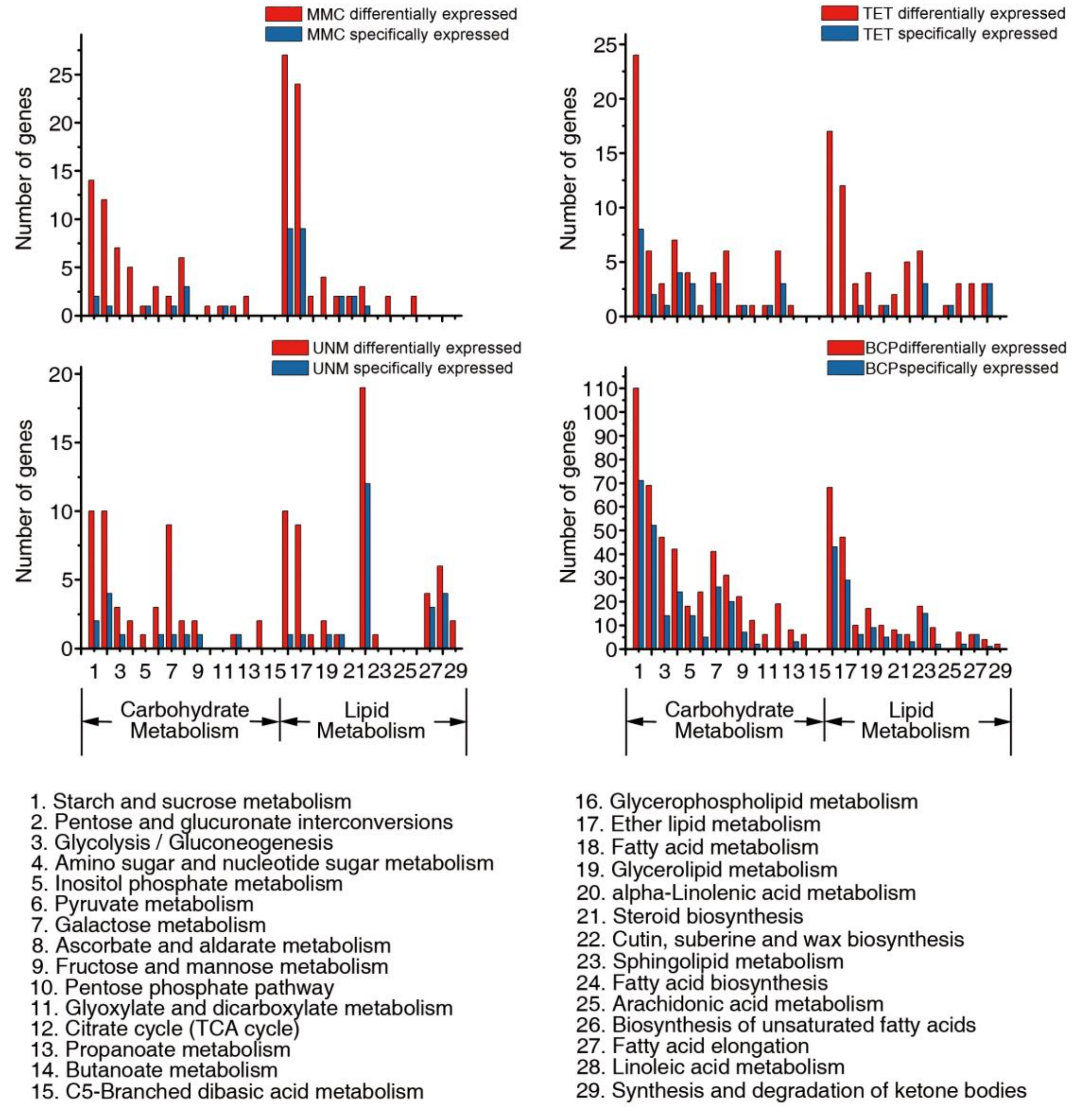

Supplementary Fig. S6 KEGG pathway enrichment analysis of differentially expressed and stage-specifically expressed genes.

The bar graph highlighting the distribution of the differentially expressed and stage-specifically expressed genes at individual stages into pathway categories based on KEGG pathway annotations.
